# Multimodal MRI-Based Study in Patients with *SPG4* Mutations

**DOI:** 10.1371/journal.pone.0117666

**Published:** 2015-02-06

**Authors:** Thiago J. R. Rezende, Milena de Albuquerque, Gustavo M. Lamas, Alberto R. M. Martinez, Brunno M. Campos, Raphael F. Casseb, Cynthia B. Silva, Lucas M. T. Branco, Anelyssa D'Abreu, Iscia Lopes-Cendes, Fernando Cendes, Marcondes C. França

**Affiliations:** 1 Departament of Neurology, University of Campinas (UNICAMP), Campinas, São Paulo, Brazil; 2 Department of Medical Genetics, University of Campinas (UNICAMP), São Paulo, Campinas, Brazil; University of Ulm, GERMANY

## Abstract

Mutations in the *SPG4* gene (SPG4-HSP) are the most frequent cause of hereditary spastic paraplegia, but the extent of the neurodegeneration related to the disease is not yet known. Therefore, our objective is to identify regions of the central nervous system damaged in patients with SPG4-HSP using a multi-modal neuroimaging approach. In addition, we aimed to identify possible clinical correlates of such damage. Eleven patients (mean age 46.0 ± 15.0 years, 8 men) with molecular confirmation of hereditary spastic paraplegia, and 23 matched healthy controls (mean age 51.4 ± 14.1years, 17 men) underwent MRI scans in a 3T scanner. We used 3D T1 images to perform volumetric measurements of the brain and spinal cord. We then performed tract-based spatial statistics and tractography analyses of diffusion tensor images to assess microstructural integrity of white matter tracts. Disease severity was quantified with the Spastic Paraplegia Rating Scale. Correlations were then carried out between MRI metrics and clinical data. Volumetric analyses did not identify macroscopic abnormalities in the brain of hereditary spastic paraplegia patients. In contrast, we found extensive fractional anisotropy reduction in the corticospinal tracts, cingulate gyri and splenium of the corpus callosum. Spinal cord morphometry identified atrophy without flattening in the group of patients with hereditary spastic paraplegia. Fractional anisotropy of the corpus callosum and pyramidal tracts did correlate with disease severity. Hereditary spastic paraplegia is characterized by relative sparing of the cortical mantle and remarkable damage to the distal portions of the corticospinal tracts, extending into the spinal cord.

## Introduction

Hereditary spastic paraplegias (HSP) are a clinically and genetically heterogeneous group of neurodegenerative disorders characterized by progressive spasticity and weakness of the lower limbs [1]. These features are related to the degeneration of long neurons in the corticospinal tract [1,2]. HSP can be classified into “pure” or “complicated” forms, depending, respectively, on the absence or the presence of other neurological or systemic abnormalities, such as dementia, cognitive impairment, ataxia and epilepsy [1]. HSP can be inherited as an autosomal dominant, autosomal recessive or X-linked disorder [3].

Mutations in the *SPG4* gene which encodes spastin, a protein of the AAA family of proteins [1] are the most frequent cause of autosomal dominant HSP (ADHSP) and are responsible for 35–45% of all patients [4]. SPG4-HSP is considered a “pure” form of HSP, but urinary problems and decreased sense of vibration in the lower limbs are frequent findings. Nevertheless, mental retardation, dementia and muscle wasting may be present, especially in patients with longer disease duration [1]. The widely variable clinical presentation of patients with SPG4-HSP suggests that damage is not restricted to neurons in the corticospinal tract, and might also be found in other areas of the central nervous system (CNS) [1,5]. Despite that, very little is known about the extension of encephalic and spinal damage in patients with SPG4-HSP; only a few studies with small cohorts of patients have been carried out so far [5].

In this scenario, we designed a MRI-based study to systematically investigate cerebral and spinal cord (SC) damage in patients with SPG4-HSP. To investigate possible gray matter damage, we used the FreeSurfer software (*https://surfer.nmr.mgh.harvard.edu/‎*). This is a robust and specific tool to evaluate cerebral cortex, which has been successfully used in similar neurodegenerative diseases [6,7]. In addition, we employed diffusion tensor images (DTI) to characterize microstructural white matter involvement in the disease. Finally, we assessed spinal cord damage through MRI-based quantitative analyses of the cervical region. Therefore, our main objective was to determine the regions of the central nervous system involved in SPG4-HSP and to evaluate whether these structural parameters correlate with clinical data.

## Methods

### Subject Selection

We initially recruited 13 patients with molecular confirmation of SPG4-HSP, but 2 of them were removed from further analyses due to MRI abnormalities not related to the disease itself: one because of widespread white matter damage (microvascular disease) and the other with congenital hydrocephalus. Therefore, eleven patients (Table 1) were included in the final MRI analyses (mean age 46.0 ± 15.0 years, 8 men). These subjects were recruited from the Neurology Outpatient Clinic at UNICAMP hospital between 2012 and 2014. None of them had other associated neurological disorders. Severity of the disease was quantified using the Spastic Paraplegia Rating Scale (SPRS) [8]. Molecular diagnosis was performed according to methods previously described [4]. Imaging findings were compared with a group of 23 age and gender matched healthy controls (mean age 51.4 ± 14.1 years, 17 men). All controls had normal neurologic examination and MRI scans, none of them had family history of neurological disease. None of the images from patients and controls had significant motion artifacts.

**Table 1:** Characterization of the Cohort.

Patient	Age (years)	Disease Duration (years)	Gender	SPRS	Mutation	Mutation Type
1	53	20	M	21	c.1255G>T	Nonsense
2	16	6	M	5	c.1255G>T	Nonsense
3	46	10	F	8	c.1378C>T	Missense
4	40	10	M	8	c.1651G>C	Missense
5	60	25	M	29	c.1651G>C	Missense
6	32	8	M	11	c.1651G>C	Missense
7	62	23	F	34	c.1651G>C	Missense
8	54	30	M	21	c.1667delCA	Frameshift deletion
9	66	10	F	15	c.1535delA	Frameshift deletion
10	36	4	M	17	c.1495C>T	Missense
11	58	7	M	13	c.1495C>T	Missense

Clinical and genetic data of patients with SPG4-HSP.

Our institution ethics committee, Comitê de Ética em Pesquisa da Faculdade de Ciências Médicas—UNICAMP, approved this study and written informed consent was obtained from all participants.

### MRI acquisition protocol

All patients and controls underwent high resolution MRI on a 3T Achieva-Intera PHILLIPS Scanner. Routine T1 and T2 weighted sequences were performed for all subjects to exclude unrelated abnormalities.

For Freesurfer and Spineseg analyses, we used volumetric T1 images of the brain and spinal cord acquired using a standard 8 channel head coil: sagittal orientation, voxel matrix 240x240x180, voxel size 1x1x1mm3, TR/TE 7/ 3.201ms, flip angle 8°.

For DTI analyses, we used a Spin echo DTI sequence: 2x2x2 mm^3^ acquiring voxel size, interpolated to 1x1x2 mm^3^; reconstructed matrix 256x256; 70 slices; TE/TR 61/8500 ms; flip angle 90°; 32 gradient directions; no averages; max b-factor = 1000 s/mm^2^; six minutes scan.

### Cortical thickness analyses

The Freesurfer software v.5.3 was used to measure cortical thickness and volume in this study. Initially, MRI images underwent the following steps: correction for magnetic field inhomogeneity; alignment to a specific atlas; skull removal; and the segmentation of the voxels into GM, WM and CSF [9,10]. Cortical thickness was then calculated based in the shortest distance of two surfaces created from the processed voxels: the white one, which is the interface between GM and WM, and the pial one. A Gaussian filter of 10 mm FWHM was used for smoothing the surface in all analyses. Estimated Intracranial Volume (eTIV) and the volume of subcortical structures were also recorded [11]. All processed images were checked for possible errors in segmentation or CSF boundary and if required, a manual intervention was done. However, this was not necessary in any of the images.

Cortical thickness abnormalities between the patient and control groups were evaluated using a General Linear Model (GLM) using age, gender and eTIV as covariates. The eTIV was used as a covariate because it was slightly different between controls and patients (p-value < 0.001). In order to correct for multiple comparisons, we employed Monte Carlo simulations, which enables the identification of significant vertex-wise group clusters. This analysis was performed using the Qdec suite running in Freesurfer (https://surfer.nmr.mgh.harvard.edu/fswiki/FsTutorial/QdecGroupAnalysis_freeview).

In addition to vertex-wise comparisons, FreeSurfer enables the comparison of cortical thickness and subcortical volume measurements for parcellation [11,12] taking into account anatomical atlases such as proposed by Desikan [12]. The statistical analysis was done using another GLM with age, gender and eTIV as covariates to assess cortical thickness and subcortical volume differences between the two groups for each region. All results were corrected for multiple comparisons using the Dunn-Sidak correction (level of significance α = 0.001). This analysis was performed using the SYSTAT software v13.0 (San José, CA).

### Tract based spatial statistics

The FMRIB toolbox on FSL v.4.1.4 was used to create maps of fractional anisotropy (FA), mean diffusivity (MD), radial diffusivity (RD) and axial diffusivity (AD). These were used in the comparison between patients and controls, which were performed using the tract based spatial statistics (TBSS) algorithm on FSL v4.1.4 [13].

The following TBSS steps preceded the statistical analyses: FA images were aligned to each other using the nonlinear registration; they were then averaged and a mean FA image was created; this enabled the generation of a mean FA skeleton, which represents all “shared” tracts among the subjects in the study. Next, the preprocessed FA image of each patient was “projected” onto the mean FA skeleton. To visualize the statistical maps of MD, AD and RD, these parameters were applied over the mean FA skeleton.

For the statistical analysis, a two-sample t-test was used to search for differences between patients and controls in FA, MD, AD and RD parameters. We used a cluster based correction for multiple comparisons (p-value<0.05). In order to investigate possible correlations between TBSS results and clinical data, we employed a general linear model and applied a TFCE to correct for multiple comparisons. We used the Johns Hopkins white matter DTI based atlas, available in the FSL software, to identify white matter tracts with abnormal findings.

### Tractography

The pyramidal tracts of each subject were studied due to its relevance to the pathology. The tensor calculation of all images was performed using the ExploreDTI software (A. Leemans, University Medical Center, Utrecht, The Netherlands) and the subsequent fiber tractography through a unbiased semiautomatic deterministic methodology briefly described below [14]. Basically, regions of interest (ROIs) to seed the tract were manually drawn on a normalized template. This template was created with non-diffusion weighted images of 10 Brazilian control subjects (mean age = 33 years; age range = 22–47 years; 50% women). Sequentially, the method uses the 3D deformation fields matrix of each subject to apply an inverse normalization operation (SPM8-Deformation fields algorithm), using the variants between native and standardized space to bring the normalized ROIs to that subject specific space. Finally, the adjusted (native space) ROIs were used as a strategy to the fiber tracking.

The fiber tracking parameters were set the same for all subjects: minimal FA to start track = 0.25; minimal FA to keep tracking = 0.25; maximal tract angle = 20° minimal fiber length = 10 mm. The resultant tracts were visually checked and the average FA, AD and RD were separately calculated for each hemisphere. The diffusion values were estimated by averaging over all voxels in a given tract. We also divided patients into two groups based on genetic features (nonsense/frameshift mutations vs missense mutations) and compared tractography results of both with a two-sample t-test. This analysis was not corrected for multiple comparisons because of the reduced number of repeated tests. All statistical analyses were performed in Systat v.13.0 software.

### Spinal cord morphometry

The Spineseg software was used to estimate cervical spinal cord (SC) area and eccentricity [15]. This tool resamples the MR images correcting variations in imaging angle and neck position. Next, it segments semi-automatically the cross-sections of the spinal cord and fits an ellipse to the segmentation region. SpineSeg requires that the user insert a few nodes following the spine and a single point inside the spinal cord. SpineSeg carries out measures of spinal cord area (CA) and spinal cord eccentricity (CE). These measures were performed at the higher section of the intervertebral disc between C2 and C3. Then, CA was the area of spinal cord on the selected slice expressed in mm^2^. The CE was calculated analytically from the better ellipse which fitted in the cord segmentation [16]. Furthermore, CE was taken as an estimate of the antero-posterior flattening of the cord in our patients. All measurements were performed by a single investigator (LMTB) who was blind for the clinical status of the patients. The statistical analyses were done using ANCOVA with age, gender and eTIV as covariates. The level of significance was adjusted for 0.05, corrected for multiple comparisons. All statistical analyses were performed in Systat v.13.0 software.

### Correlation Analyses

Regions found to be atrophic or with abnormal tractography results in patients with SPG4-HSPwere correlated with clinical data (disease duration and SPRS) through Pearson correlation coefficients. All results were corrected for multiple comparisons, Dunn-Sidak correction. All statistical analyses were performed in Systat v.13.0 software.

## Results

### Between-group comparisons

**FreeSurfer**. Cortical and subcortical analyses using the FreeSurfer package did not show cortical thinning or volumetric reduction at any structure after correction for multiple comparisons. We highlight these results by showing data for the motor regions in S1 Table.

**Fig 1:**
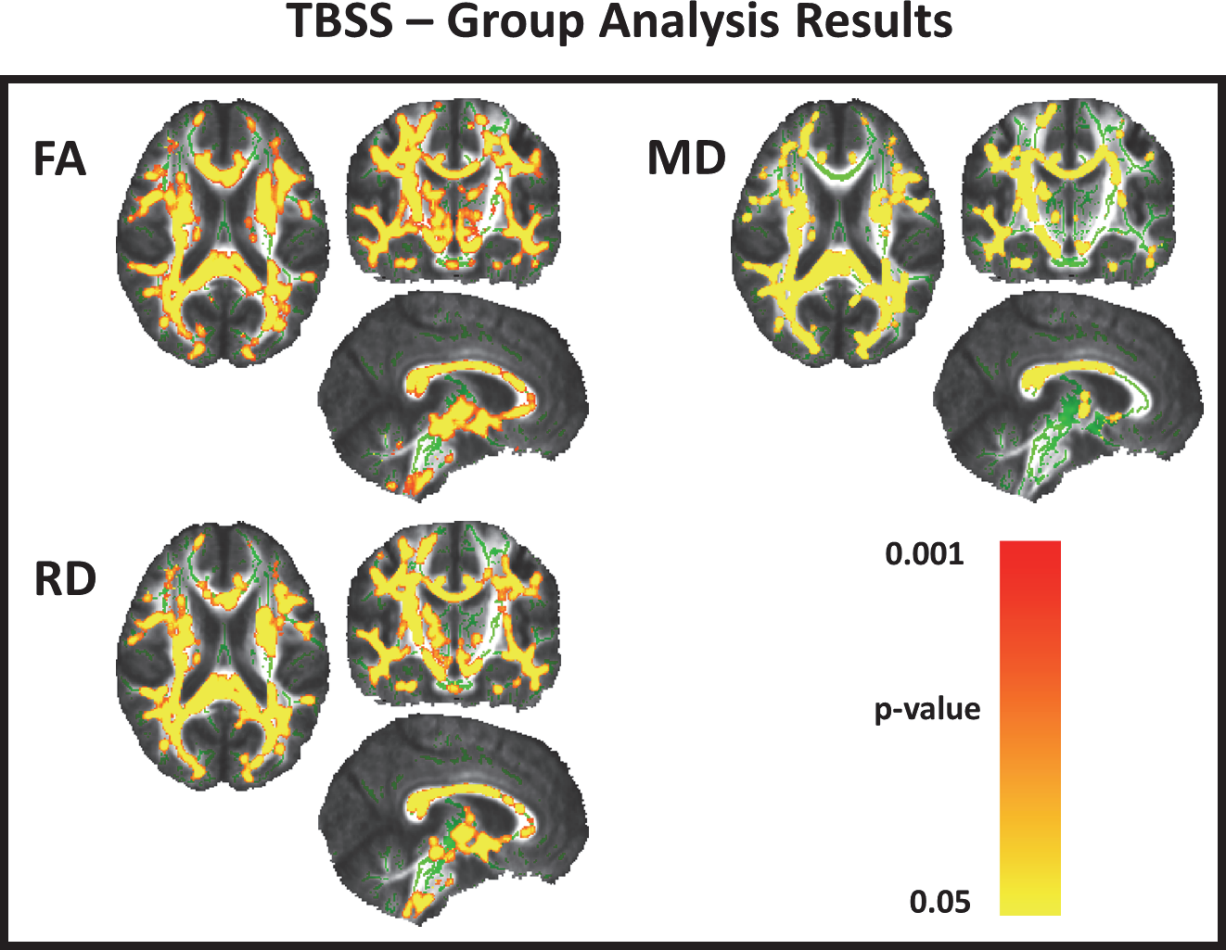
TBSS analyses showing microstructural damage in SPG4-HSP. TBSS results showing areas of reduced FA and increased MD, RD and AD in patients with SPG4 mutations after comparison with age and sex matched controls. Areas with reduced FA and increased MD, RD and AD are shown in yellow-red and represent cluster based values (p<0.05, corrected). Results are shown on the MNI152 1 mm template.

**TBSS**. TBSS identified extensive microstructural damage in patients with SPG4-HSP. We identified reduced FA as well as increased MD and RD in the corticospinal tracts, posterior cingulate gyri, posterior subcortical regions and the splenium of corpus callosum (CC) (Fig. 1).

**Tractography**. Automatic tractography showed significantly reduced FA values at the corticospinal tracts on both sides and on average in the SPG4-HSP group (Table 2). We did not find differences between the groups regarding MD, RD and AD values. Furthermore, patients with nonsense/frameshift mutations (mean age and duration: 41.5 ± 18.2 and 17.0 ± 11.5 years, respectively) presented significantly smaller FA in right and left corticospinal tracts compared to those patients with missense mutations (mean age and duration: 49.0 ± 13.5 and 12.7 ± 9.0 years, respectively) (Table 3).

**Table 2:** Tractography Results.

FA values			
Corticospinal Tract	Control (mean±SD)	Patient (mean±SD)	p-value
Left Hemisphere	0.653701 ± 0.021689	0.622842 ± 0.031106	0.002
Right Hemisphere	0.642387 ± 0.030750	0.608979 ± 0.045752	0.019
Average	0.600654 ± 0.021279	0.582428 ± 0.019791	0.028
**MD values**			
Left Hemisphere	0.000781 ± 0.000034	0.000783 ± 0.000047	0.878
Right Hemisphere	0.000795 ± 0.000040	0.000798 ± 0.000041	0.825
Average	0.000744 ± 0.000019	0.000748 ± 0.000018	0.519
**AD values**			
Left Hemisphere	0.001451 ±0.000069	0.001409 ± 0.000072	0.125
Right Hemisphere	0.001456 ± 0.000072	0.001412 ± 0.000068	0.111
Average	0.001318 ± 0.000044	0.001302 ± 0.000041	0.343
**RD values**			
Left Hemisphere	0.000446 ± 0.000028	0.000470 ± 0.000044	0.065
Right Hemisphere	0.000464 ± 0.000042	0.000491 ± 0.000052	0.119
Average	0.000458 ± 0.000020	0.000472 ± 0.000017	0.052

Results of automatic tractography for the corticospinal tracts in patients and controls.

**Table 3:** Neuroimaging findings according to *SPG4* mutation subtypes.

Corticospinal Tract			
	Nonsense/Frameshift(mean±SD)	Missense (mean±SD)	p-value
FA Average	0.572 ± 0.015	0.589 ± 0.021	0.094
FA Right Hemisphere	0.581± 0.030	0.628 ± 0.046	0.043
FA Left Hemisphere	0.600 ± 0.026	0.638 ± 0.026	0.029
**Cervical Spinal cord**			
Area	54.725 ± 4.305	61.680 ± 8.054	0.058

Group analysis based on genetic features, Nonsense/Frameshift mutation vs Missense mutation.

**Fig 2:**
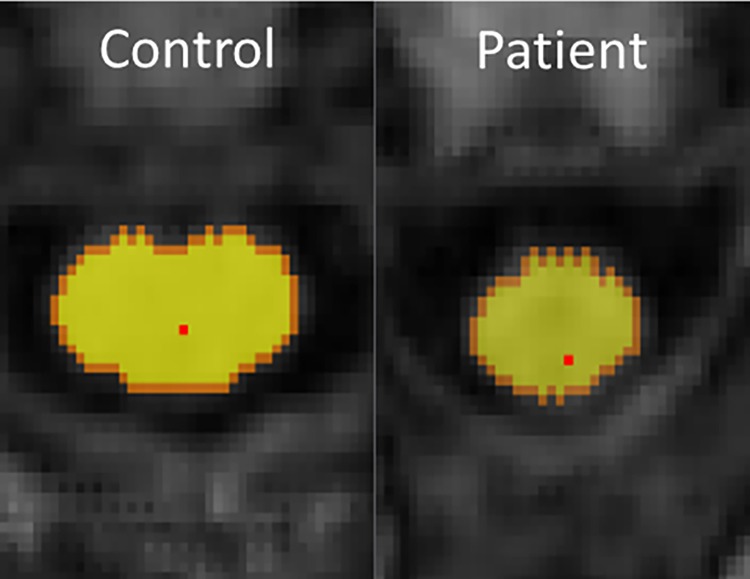
Segmentation of spinal cord using Spineseg software. Spineseg layout showing the segmentation of the cervical spinal cord at C2-C3 level in a control and a patient with SPG4-HSP.

**SpineSeg**. The SpineSeg package showed spinal cord atrophy in SPG4-HSP patients (mean controls = 68.04 ± 9.05 mm^2^; mean patients = 58.90 ± 7.42 mm^2^; p-value = 0.025) (Fig. 2). However, we did not find flattening of spinal cord, given by the eccentricity, (mean controls = 0.7675 ± 0.0557; mean patients = 0.7693 ± 0.0718; p-value = 0.893).

### Correlation Analyses

**Fig 3:**
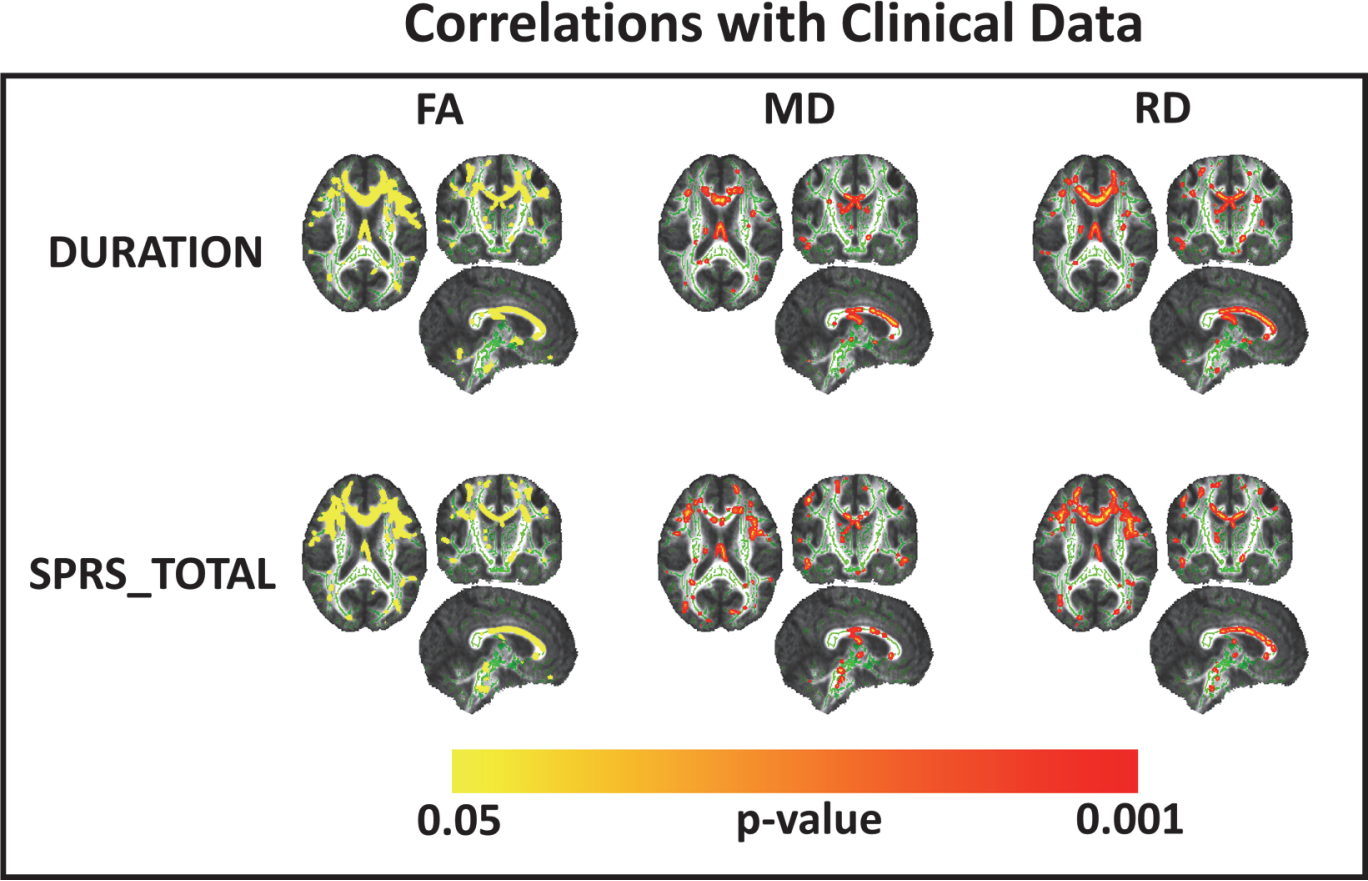
Correlation Analyses of TBSS results and clinical data. Upper lane—TBSS results showing white matter regions that presented significant negative correlation between disease duration and DTI parameters (first column—FA, Second column—MD, third column—RD). Lower lane—TBSS results showing white matter regions that presented significant negative correlation between Spastic paraplegia rating scale scores and DTI parameters (first column—FA, Second column—MD, third column—RD). Results are shown on the MNI152 1 mm template.

We found significant correlation between clinical data and DTI parameters (FA, MD and RD) using TBSS approach (Fig. 3). In contrast, we did not find any correlation between clinical data with measures from tractrography. However, we found a significant correlation between FA for the right corticospinal tract and area of the spinal cord (r = 0.637, p = 0.048).

## Discussion

Comprehensive neuroimaging studies have proven useful to understand the pathophysiology of several heredodegenerative disorders. However, this is especially challenging in the case of rare monogenic disorders such as HSPs. In SPG4-HSP, there are few previous image-based studies with small cohorts, but none of them employed a multimodal MRI-approach [2,17–19]. In addition, in some of these studies patients with distinct HSP genetic subtypes were combined into a single analysis increasing the possibility of introducing confounding factors as a result of genetic and clinical heterogeneity [20,21]. To overcome these limitations, we performed a comprehensive neuroimaging study in a large cohort of patients with molecular confirmation of SPG4/Spastin-related hereditary spastic paraplegia. Our results indeed show that damage in SPG4-HSP mostly involves the distal portions of the corticospinal tracts (extending into the spinal cord) and relatively spares the cortical mantle.

Our results support the current pathophysiological model of SPG4-HSP as a distal axonopathy. Spastin, the protein encoded by the *SPG4* gene, is a member of the ATPases associated with various cellular activities [22]. One of the most significant is the microtubule-severing function, which is important for membrane trafficking of organelles to reach the distal axon [22]. From a DTI point of view, our finding of increased RD with “normal”AD suggests that corticospinal tract damage is demyelinating in SPG4-HSP, which is in apparent contrast to motor evoked potential studies, which show a predominantly axonal pattern [23]. However, we must take into account that MEP is a measure that relies upon structural integrity of the whole corticospinal tract (all the way from the lower cord up to the cortex). In contrast, our DTI results are restricted to the upper portions of the corticospinal tract. We believe that the histological abnormalities in the proximal and distal portions of the corticospinal tracts are different in SPG4-HSP (since this is a dying-back axonopathy), and this might explain the different pattern of abnormalities related to water diffusion. In particular, we hypothesize that the cellular abnormalities that occur in the soma and proximal axons of a degenerating fiber (eg, accumulation of organelles and proteins) might difficult the longitudinal motion of water molecules along the axon. This would explain the lack of AD abnormalities in cerebral CST [24, 25].

There are only 2 pathological reports of patients with molecular confirmation of SPG4-HSP [26,27]. Both studies, however, emphasized spinal cord damage involving lateral and posterior columns as the major macroscopic finding. These findings are in agreement with our imaging findings that identified cervical cord atrophy without flattening. Hedera et al found similar results in a study with a mixed group of pure HSP patients [21]. Such pattern has been found in disorders characterized by preferential damage to the anterolateral portions of the SC, which harbors the corticospinal tracts [28]. All these findings are in consonance with the main phenotypical expression in SPG4-HSP patients, that is pure spastic paraparesis with slightly decreased vibration sense and urinary urgency.

DTI-based analyses identified extensive WM microstructural damage in SPG4-HSP. Corticospinal tracts, posterior cingulate gyri and the splenium of CC were particularly affected. Garaci*et al*. performed TBSS analyses in three patients with SPG4-HSP and also found reduced FA in the posterior region of the CC [20]. They failed to identify corticospinal tract damage, but this was probably due to the small sample size. Our TBSS results are very similar to those reported by Duning et al who used a different approach (voxel-based FA analysis) in a cohort of 6 patients. In contrast to these studies, however, we extended our analyses to evaluate axial (AD), radial (RD) and mean diffusivity (MD) in addition to fractional anisotropy (FA) and our results are in agreement with a study carried out by Aghakhanyan et al. which also found abnormalities at FA, MD and RD, without significant results at AD [29]. The extensive WM abnormalities found in non-motor regions (eg, posterior cingulate gyri and the splenium of corpus callosum) might be associated with cognitive dysfunction in these subjects. None of our patients had overt cognitive deficits or complaints, but we did perform a comprehensive neuropsychological evaluation to address this hypothesis. Further studies are needed to investigate the association of cognitive impairment and WM damage in SPG4-HSP.

We attempted to compare WM abnormalities in patients with distinct types of *SPG4* mutations. Interestingly, those with missense mutations presented higher FA at the both right and left CST in comparison to those with nonsense and frameshift mutations. This is particularly relevant because both subgroups have similar mean age and disease duration. Therefore, it is possible that severity of structural abnormalities depends on the mutation of each subject (and its impact on Spastin structure and/or expression). These results are in line with Karle et al who found distinct neurophysiological patterns in patients with missense vs non-misense SPG4 mutations [23].

We did not find GM damage in our patients, which is in accordance with the usual phenotype of SPG4-HSP as a pure HSP. This is in striking contrast to the volumetric findings reported in complicated HSPs, particularly SPG11-HSP. In the latter, there is widespread GM atrophy (particularly at the basal ganglia), which explains the more heterogeneous phenotype that frequently includes parkinsonism and dystonia [6]. Our results are also quite different from those reported in patients with ALS, which is the prototypical disease of upper motor neurons. Although microstructural abnormalities in the corticospinal tracts are seen in both ALS and SPG4-HSP, cortical thinning at precentral cortices is only observed in the former [30]. These findings strongly support the concept that both diseases have different mechanisms, and that SPG4-HSP is a dying-back axonopathy rather than a primary neuronopathy.

TBSS analyses revealed WM abnormalities that correlated with disease duration and severity. These were mostly located at the corpus callosum and to a much lesser extent at the CST. These findings suggest that DTI-based parameters of the CC and CST might be useful as prognostic neuroimaging markers in SPG4-HSP. Unexpectedly, there was no correlation between clinical parameters and tractrography data. These conflicting results can be explained by the distinct approaches used in each method—tractrography results are actually the mean value of all voxels in the tract, whereas TBSS performs na individual assessment for each voxel in the tract. Therefore, the first one might attenuate any significant focal abnormality within a specific tract. In addition, we identified a significant correlation between corticospinal tract FA and cervical cord area. This finding supports the concept of CST damage as the anatomical substrate for cord atrophy in SPG4-HSP. It also suggests that CST damage at the brain and at the spinal cord follow a parallel course.

## Conclusion

The present study supports the concept of SPG4-HSP as a distal motor axonopathy, and points toward the involvement of non-motor areas as well. DTI-based analyses are promising to assess the neurodegeneration associated to SPG4-HSP.

## Supporting Information

S1 Table FreeSurfer measurements. Comparison of cortical thickness measurements for motor regions between controls and patients with SPG4-HSP.
(DOCX)
