# Supplementary material for: Multimodal MRI-Based Study in Patients with SPG4 Mutations
Source: PLoS One. 2015 Feb 6;10(2):e0117666. doi: 10.1371/journal.pone.0117666 (PMC4320056; doi:10.1371/journal.pone.0117666)
Supplement: S1 Table — Comparison of cortical thickness measurements for motor regions between controls and patients with SPG4-HSP. (DOCX) [file pone.0117666.s001.docx]

**Supporting Information**

**S1Table.**

**FreeSurfer measurements.** Comparison of cortical thickness measurements for motor regions between controls and patients with SPG4-HSP.

|  | **Left-Hemisphere** | | | **Right- Hemisphere** | | |
| --- | --- | --- | --- | --- | --- | --- |
| **Structure** | **Control mean (mm)** | **SPG4 mean (mm)** | **p-value** | **Control mean (mm)** | **SPG4 mean (mm)** | **p-value** |
| **Postcentral Gyrus** | 2.004 ± 0.160 | 1.888 ± 0.191 | 0.483 | 1.904 ± 0.201 | 1.797 ± 0.154 | 0.359 |
| **Precentral Gyrus** | 2.619 ± 0.252 | 2.490 ± 0.197 | 0.777 | 2.583 ± 0.229 | 2.446 ± 0.183 | 0.574 |
| **Central Sulcus** | 1.738 ± 0.117 | 1.630 ± 0.129 | 0.157 | 1.703 ± 0.122 | 1.595 ± 0.098 | 0.131 |
| **Inferior Part of Precentral Sulcus** | 2.258 ± 0.182 | 2.218 ± 0.202 | 0.452 | 2.249 ± 0.191 | 2.157 ± 0.150 | 0.524 |
| **Superior Part of Precentral Sulcus** | 2.167 ± 0.231 | 2.053 ± 0.168 | 0.305 | 2.141 ± 0.154 | 2.016 ± 0.163 | 0.110 |
